# Comparing Bacterial Community Composition of Healthy and Dark Spot-Affected *Siderastrea siderea* in Florida and the Caribbean

**DOI:** 10.1371/journal.pone.0108767

**Published:** 2014-10-07

**Authors:** Christina A. Kellogg, Yvette M. Piceno, Lauren M. Tom, Todd Z. DeSantis, Michael A. Gray, Gary L. Andersen

**Affiliations:** 1 U.S. Geological Survey, St. Petersburg Coastal and Marine Science Center, St. Petersburg, Florida, United States of America; 2 Lawrence Berkeley National Laboratory, Berkeley, California, United States of America; 3 Second Genome, Inc., San Bruno, California, United States of America; Missouri University of Science and Technology, United States of America

## Abstract

Coral disease is one of the major causes of reef degradation. Dark Spot Syndrome (DSS) was described in the early 1990's as brown or purple amorphous areas of tissue on a coral and has since become one of the most prevalent diseases reported on Caribbean reefs. It has been identified in a number of coral species, but there is debate as to whether it is in fact the same disease in different corals. Further, it is questioned whether these macroscopic signs are in fact diagnostic of an infectious disease at all. The most commonly affected species in the Caribbean is the massive starlet coral *Siderastrea siderea*. We sampled this species in two locations, Dry Tortugas National Park and Virgin Islands National Park. Tissue biopsies were collected from both healthy colonies and those with dark spot lesions. Microbial-community DNA was extracted from coral samples (mucus, tissue, and skeleton), amplified using bacterial-specific primers, and applied to PhyloChip G3 microarrays to examine the bacterial diversity associated with this coral. Samples were also screened for the presence of a fungal ribotype that has recently been implicated as a causative agent of DSS in another coral species, but the amplifications were unsuccessful. *S. siderea* samples did not cluster consistently based on health state (i.e., normal versus dark spot). Various bacteria, including Cyanobacteria and *Vibrios*, were observed to have increased relative abundance in the discolored tissue, but the patterns were not consistent across all DSS samples. Overall, our findings do not support the hypothesis that DSS in *S. siderea* is linked to a bacterial pathogen or pathogens. This dataset provides the most comprehensive overview to date of the bacterial community associated with the scleractinian coral *S. siderea*.

## Introduction

Diseases of reef-building corals are now considered a major cause of global coral reef ecosystem decline [1], [2]. The past two decades have seen a dramatic increase in the number of reports of coral diseases, particularly in the Caribbean [3]–[5]. Most of these diseases are known or suspected to be microbial in origin [6]. Moreover, microbiology is a key part of coral biology, in the same way that human microbiome studies are revealing microbes to be a critical part of human biology [7].

*Siderastera siderea*, also known as the massive starlet coral, is a common component of Caribbean reefs, occurring from the Gulf of Mexico to South America [8]. However, there has been little attention focused on the bacterial communities associated with this coral, other than one culture-based study [9], two clone library studies focused specifically on black band lesions [10], [11] and a recent pyrosequencing study of a white plague-like disease [12].

Dark spot syndrome (DSS) was first reported as a discoloration observed on *S. siderea*, *Stephanocoenia intercepta*, *Porites astreoides*, and *Montastraea cavernosa* near Columbia in the early 1990's [13]. Dark spot lesions are described as purple, black, or brown discolored areas of tissue that may be circular, elongate, ring-shaped, or occur lining the coral tissue-algal boundary of an older lesion [14]–[17] (Fig. S1). There is some argument in the literature as to whether to define dark spot as a disease (DSD) or a syndrome (DSS) [18]. This tissue discoloration has been linked to both physical [14] and microbiological [19], [20] causes, leading some to suggest it may be a non-specific stress response [14], [21]. Unlike coral diseases such as black band or white plague, dark spot lesions rarely cause whole colony mortality and result in relatively low net tissue loss [22]. Further, dark spot lesions have been observed to disappear in as little as a month [21], [22]. It has been suggested that dark spots may signal different conditions in different coral species [20], [22]–[24]. Given that breadth of scope, we have opted to use the more comprehensive term ‘dark spot syndrome’ (DSS), but acknowledge that it can be regarded as synonymous with DSD given the recent push to apply a broader medical definition (“any inhibition of normal function”) to coral diseases [18], [25].

Although DSS has been identified in several species of Caribbean corals, *S. siderea* is the most frequently affected [14], [24], [26]–[29]. Previous coral disease work has shown that bacterial communities shift when their hosts are stressed (even in normally pigmented tissues [30]). Therefore, we hypothesized that there would be a shift in the bacterial communities of DSS-affected tissues compared to healthy colonies, regardless of whether DSS was due to a general immune response or an infection. Because there appear to be multiple possible etiologies for DSS lesions, we wondered if there would be geographic differences or perhaps multiple clusters of DSS bacterial communities rather than the single diagnostic grouping we observed previously in a white plague-like disease [31]. To address these questions, we collected *S. siderea* samples from Florida and the Virgin Islands and used PhyloChip G3 DNA microarrays to examine the breadth of taxonomic diversity of the bacterial communities associated with both healthy and DSS-affected colonies. This is the first study to apply molecular techniques to the study of DSS in *S. siderea*.

## Materials and Methods

### Ethics Statement

These collections were made under permits VIIS-2008-SCI-0033 (study VIIS-08033) and DRTO-2009-SCI-0018 (study DRTO-00074), granted to the first author by the Virgin Islands National Park and Dry Tortugas National Park, respectively. No ethical approval was required for the experimental research described here.

### Sample sites and collections

**Figure 1:**
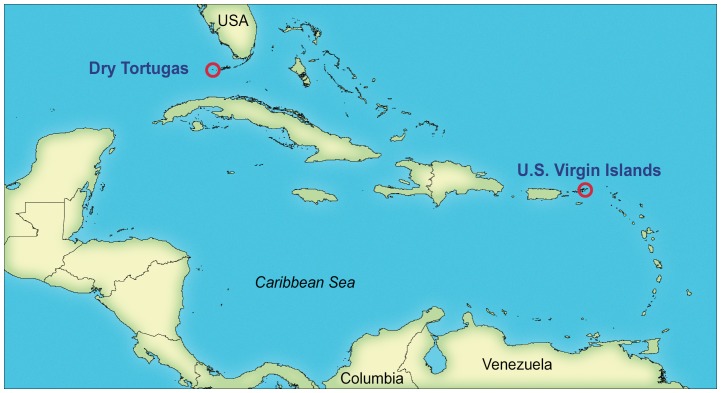
Map of Florida and the Caribbean showing the two sampling locations.

Healthy (i.e., exhibiting normal pigmentation) and DSS-affected *S. siderea* samples were collected from Dry Tortugas (DRTO) and Virgin Islands (VIIS) National Parks during the summer of 2009 (Fig. 1). The collection areas at each park were localized such that most coral colonies sampled were within 10 meters of another, and the maximum distance between two coral colonies sampled at one site did not exceed 140 meters. Five healthy and five DSS-affected colonies were sampled from the three-lobed patch reef in Hawksnest Bay (18°20′50 N, 64°46′50 W), St. John, VIIS on June 23, 2009 (Table 1). The five healthy colonies were all located to the north (offshore) of the eastern-most lobe. The dark spot colonies were found on the eastern side of each of the three reef lobes. The water temperature was 29°C with a salinity of 34 ppt. Samples from five healthy and five DSS-affected colonies also were collected from an area on the west side of Loggerhead Key, DRTO (24°38′05 N, 82°55′22 W) on August 3–5, 2009 (Table 1). The water temperature was 31–32°C with a salinity of 35 ppt. Three of the samples, one healthy and two dark spot (DRTOSSD02, DRTOSSH05, and DRTOSSD09) were collected and their bacterial community DNA extracted, but they failed to amplify, so they were not included in Table 1 and do not appear in any subsequent analyses.

**Table 1:** *Siderastrea siderea* samples processed for microarray analysis.

Sample ID	Location	Date Collected	Health State	Depth (m)
VIISSSH01	Virgin Islands National Park	July 23, 2009	normal	3.7
VIISSSH02	Virgin Islands National Park	July 23, 2009	normal	4.3
VIISSSH03	Virgin Islands National Park	July 23, 2009	normal	4.6
VIISSSH04	Virgin Islands National Park	July 23, 2009	normal	4.6
VIISSSH05	Virgin Islands National Park	July 23, 2009	normal	4.6
VIISSSD06	Virgin Islands National Park	July 23, 2009	dark spot	3.7
VIISSSD07	Virgin Islands National Park	July 23, 2009	dark spot	3.7
VIISSSD08	Virgin Islands National Park	July 23, 2009	dark spot	4.6
VIISSSD09	Virgin Islands National Park	July 23, 2009	dark spot	4.6
VIISSSD10	Virgin Islands National Park	July 23, 2009	dark spot	3.7
DRTOSSH01	Dry Tortugas National Park	Aug 3, 2009	normal	2.1
DRTOSSH03	Dry Tortugas National Park	Aug 3, 2009	normal	1.8
DRTOSSH04	Dry Tortugas National Park	Aug 3, 2009	normal	2.1
DRTOSSH06	Dry Tortugas National Park	Aug 4, 2009	normal	2.4
DRTOSSD07	Dry Tortugas National Park	Aug 4, 2009	dark spot	2.1
DRTOSSD08	Dry Tortugas National Park	Aug 4, 2009	dark spot	0.6
DRTOSSD10	Dry Tortugas National Park	Aug 5, 2009	dark spot	3.4

Corals were sampled using a bleach-sterilized metal punch of approximately 2-cm diameter to collect two biopsies of tissue with minimal skeletal material attached. DSS-affected corals were sampled with the punch centered over discolored tissue (i.e., in the dark spot lesion). Note that we rarely encountered colonies with discolored tissue spots completely surrounded by normally pigmented tissue (samples DRTOSSD07, DRTOSSD10, VIISSSD07); instead most of our discolored tissue samples were found along the edge of larger, older lesions (Fig. S1). Coral tissue samples were immediately placed into sterile Whirl-pak bags. The punch was cleaned and re-bleached between samples to prevent any transfer of microbes or contamination. Back on shore, the coral samples were briefly rinsed with sterile-filtered seawater to remove loosely associated microbes, wrapped in sterile aluminum foil, placed into a fresh Whirl-pak bag, and flash-frozen in liquid nitrogen. Samples were transported from the field to the USGS St. Petersburg Coastal and Marine Science Center and then transferred to a −80°C freezer for storage.

### DNA extraction

In the laboratory, each pair of frozen tissue samples from a single coral colony was ground to powder using a sterile mortar and pestle on dry ice and then combined. The microbial community DNA was extracted in duplicate from each combined sample using the MO BIO PowerPlant DNA Isolation Kit with some modification as previously described by Sunagawa et al. [32]. At the end of the protocol, the replicate extractions were combined, resulting in a single microbial DNA extraction representing each coral colony sampled.

### PhyloChip G3 microarray

Microbial profiles were generated for the 17 coral samples using PhyloChip G3 microarrays as described previously [31], [33], employing the exact PCR conditions and 16S bacterial ribosomal RNA gene primers previously detailed by Kellogg et al. [31]. In some cases the primers amplified both coral and bacterial DNA [34], requiring gel extraction of the bacterial amplicons for all samples to be consistent. The difference in size between the two products (i.e., coral and bacterial amplicons) was roughly 200 base pairs (bp). To allow clear separation of the target (bacterial) amplicon, gels were run twice as long as usual (25 min) before being stopped to excise the 16S rRNA amplicon.

For each sample, 500 ng of PCR product was applied to an individual PhyloChip G3 DNA microarray. Fragmentation of the 16S rRNA gene amplicons, labeling, hybridization, staining, probe selection, probe scoring of the microarrays and initial data acquisition were conducted according to Hazen et al. [35].

### Data analyses

The taxonomy used for this dataset [36] was the same as that used for our recent study of a white plague-like disease [31] (i.e., updated since [33]). The array fluorescence intensity data were scaled to internal standards and then operational taxonomic units (OTUs) were normalized by dividing the OTU intensity value by the individual array sum and then multiplying by the average of all total array sums. Stage 1 of PhyCA analysis removed OTUs not present in any of the samples using cutoff values: q1 = 0.5, q2 = 0.93, q3 = 0.98. Post-Stage 2 (PhyCA analysis) data were obtained using cutoff values: q1 = 0, q2 = 0, q3 = 0.1. This reduced the total number of OTUs subject to analysis from roughly 60,000 (all possible OTUs) down to 9,700. OTUs only present in a single sample were removed, further decreasing the total number of OTUs to 4,978. The microarray data have been archived online at Greengenes, accessible by the following dedicated URL: (http://greengenes.lbl.gov/Download/Microarray_Data/Siderastrea_Kellogg.zip). The data also have been deposited in the NCBI's Gene Expression Omnibus [37] and are accessible through GEO Series accession number GSE60622 (http://www.ncbi.nlm.nih.gov/geo/query/acc.cgi?acc=GSE60622).

A QIIME-formatted OTU table based on the normalized intensity data and a mapping file were created and run through QIIME [38]. The script ‘summarize_taxa_through_plots.py’ was used to generate taxa summary plots of OTU relative intensity (as a proxy for relative abundance) at the phylum through family levels.

PRIMER 6 version 6.1.13 software [39] was used to conduct non-metric multidimensional scaling (NMDS) analyses. The normalized (as described above), square-root transformed intensity data also were used to calculate the two-way crossed analyses of similarity (ANOSIM) to test for significant differences in the bacterial community composition between predefined sample sets (i.e., based on collection location and health state).

### Fungal extraction and amplification

Based on recent findings by Sweet et al. [40], we also wanted to check our samples for fungal diversity. The DNA from approximately 50 mg of each powdered coral sample was extracted using the Qiagen Blood and Tissue extraction kit, following the manufacturer's Gram- positive protocol. Extractions were amplified using the nested PCR protocol described by Sweet et al. [40] with the modification that the second set of primers did not include the GC clamp that is specific to denaturing gradient gel electrophoresis (DGGE). The nested amplifications were also attempted using a different version of the ITS1 primer [41]. PCR amplicons were visualized on a 1% agarose gel.

## Results

### Bacterial diversity

**Figure 2:**
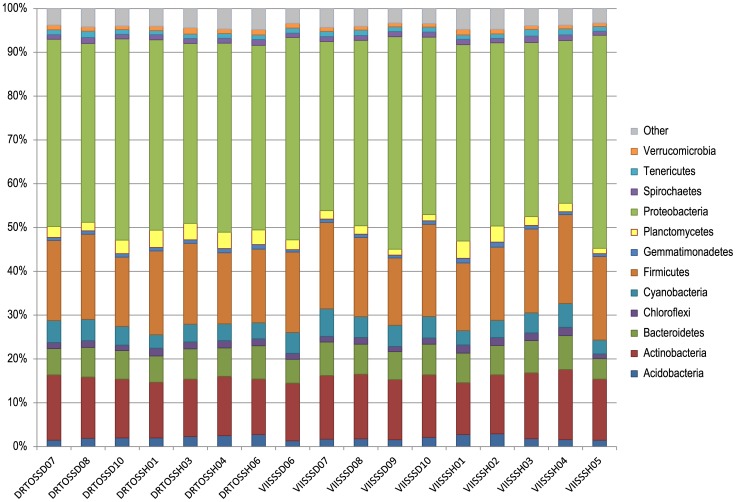
Relative diversity of the major bacterial phyla associated with *Siderastrea siderea* corals. Minor phyla (less than 0.5% of any sample) and unclassified sequences are collectively represented by the ‘other’ category.

**Figure 3:**
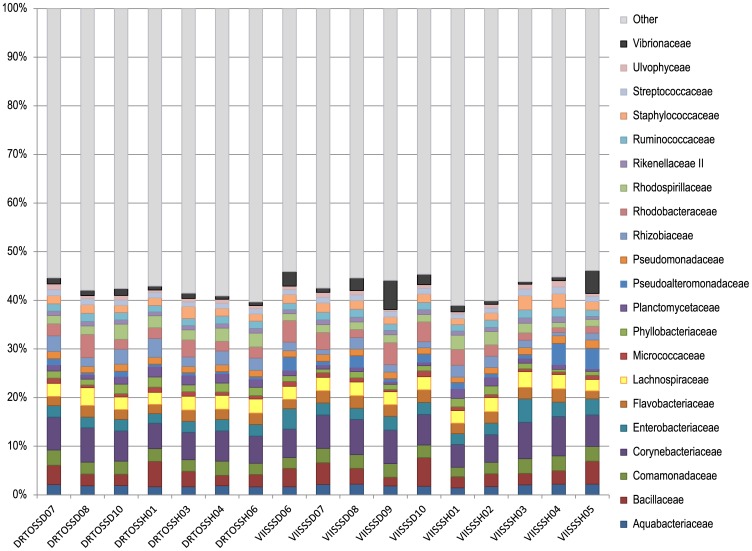
Relative diversity within bacterial families associated with *Siderastrea siderea* corals. The 21 families shown were those representing greater than 1% of at least one sample. The remaining families and unclassified sequences are collectively represented by the ‘other’ category.

The PhyloChip G3 microarrays detected 9,700 operational taxonomic units (OTUs) across the 17 samples of *S. siderea*. After removal of OTUs that only occurred in a single sample, there were 4,978 OTUs that represented 70 different phyla. The major phyla detected were Proteobacteria, Firmicutes, Actinobacteria, Bacterioidetes, Cyanobacteria, Planctomycetes, Acidobacteria, Chloroflexi, Spirochaetes, Tenericutes, Verrucomicrobia and Gemmatimonadetes (Fig. 2), partly reflecting the prominence of probes on the array for these major lineages. Regardless of health state or location (Table 1), all coral samples had similar looking bacterial communities at the phylum level (Fig. 2). When examining the samples at the family level (Fig. 3), the same similarity across location and health state was observed. The most variable family across all samples was the Vibrionaceae, which appeared slightly higher in some DSS samples from the Virgin Islands (VIISSSD06, VIISSSD08, VIISSSD09, and VIISSSD10), but also was enriched in a healthy sample (VIISSH05) (Fig. 3, Fig. S2). Less variation was observed across the other major families represented on the PhyloChip G3 and detected in these samples: Aquabacteriaceae, Bacillaceae, Comamonadaceae, Corynebacteriaceae, Enterobacteriaceae, Flavobacteriaceae, Lachnospiraceae, Micrococcaceae, Phyllobacteriaceae, Planctomycetaceae, Pseudoalteromonadaceae, Pseudomonadaceae, Rhizobiaceae, Rhodobacteraceae, Rhodospirillaceae, Rikenellaceae II, Ruminococcaceae, Staphylococcaceae, Streptococcaceae, and Ulvophyceae (Fig. 3). Over 200 additional families represented by fewer OTUs were also detected, resulting in the identification of over 600 bacterial genera (Table S1).

**Figure 4:**
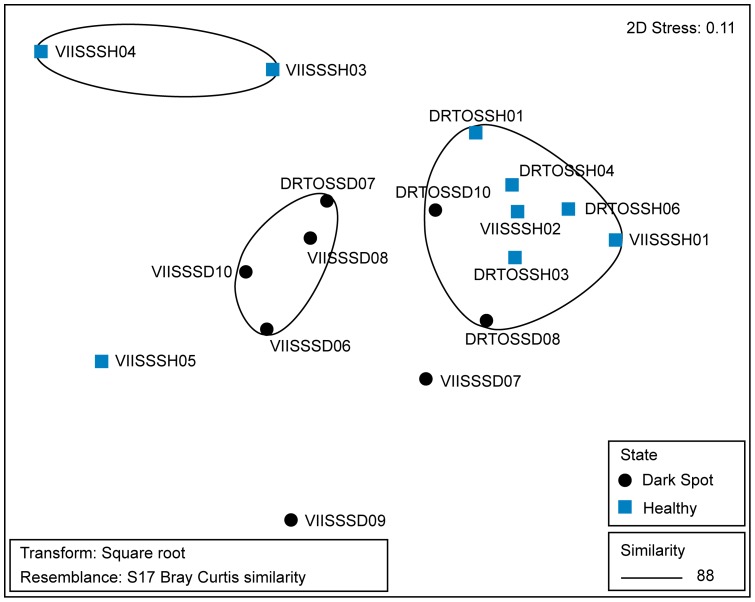
Non-metric multidimensional scaling (NMDS) diagram of the bacterial communities from each sample based on 4,978 operational taxonomic units (OTUs) detected by the PhyloChip G3 microarray. Samples with the prefix ‘DRTO’ were from Dry Tortugas National Park. Samples with prefix ‘VIIS’ were from Virgin Islands National Park.

NMDS analysis of the normalized intensity data from the 4,978 shared OTUs showed that the samples do not cluster based on geographic location (Fig. 4). Although four DSS samples did group together (Fig. 4), it was determined using a PERL script previously developed to sort microarray OTUs [31] that there were only 13 OTUs unique to that group, and none were shared by all four samples. Further, ANOSIM conducted on the 4,978 shared OTUs did not find any significant differences between either the locations (ANOSIM Global R = −0.046, p = 0.63) or health states (healthy versus DSS; ANOSIM Global R = 0.162, p = 0.067).

While there was not a single OTU or a defined group of OTUs with higher relative abundance(s) in all DSS samples compared to healthy coral samples, there were a few OTUs with notable increased relative abundance in several of the DSS samples (Figs. S2, S3, S4). For example, OTU 53657 (containing one sequence aligned with *Pseudoscillatoria coralii* str. BgP10_4S, GenBank accession FJ210722.2) had intensity values>10,000 units in four DSS samples (DRTOSSD08, VIISSSD06, VIISSSD09, VIISSSD10), whereas the intensity values for all healthy tissue samples and several other DSS samples were <1,000 units. Because *P. coralii* has been found associated with black band disease (BBD) [42]–[44] and BBD microbial consortia often include sulfate-reducing bacteria [11], [45], we also examined relative intensities of Deltaproteobacterial OTUs and appropriate OTUs in classes Clostridia and Nitrospira. No sulfate-reducing OTUs with notably higher relative intensities in DSS samples were found.

### Fungi

All attempts to amplify and clone fungal sequences from *S. siderea* samples following the method used by Sweet et al. [40] failed. The fungal primers used (ITS1F, ITS3F, ITS4F [40]) were not specific enough to differentiate between fungi and coral in *S. siderea* (similar to co-amplification of coral 18S rRNA genes by bacterial primers [34]). PCR resulted in strong amplification of a 380 bp amplicon and extremely faint to no amplification of the expected 500 bp fungal amplicon. Subsequent cloning and sequencing of the 380 bp band revealed it to be coral DNA. Attempts were made to optimize the method using gradient PCR, gel extraction and re-amplification, and employing a different fungal primer (ITS1: TCCGTAGGTGAACCTGCGG [41]). In all cases, the 380 bp coral amplicon dominated, and when present, the possibly fungal 500 bp amplicon was at such a low concentration that it was not possible to clone.

## Discussion

Opinions on the importance of DSS to reef ecology range from those considering it of limited significance due to the low mortality rates and limited tissue loss [26] to those who feel that the high frequency of occurrence and links to greater susceptibility to subsequent bleaching or disease make it a useful indicator of reef health [14], [46]. Our initial hypothesis was that we would detect differences in the bacterial communities between healthy and DSS-affected tissues as we had between healthy and white plague-like diseased corals [31], possibly with a geographic component. While half of the DSS samples formed a cluster at 88% similarity (Fig. 4), this was not statistically significant (healthy versus DSS; ANOSIM Global R = 0.162, p = 0.067), and the remaining DSS samples grouped with healthy corals or alone. We did not detect a difference in overall bacterial diversity nor relative abundance of specific taxa (as in [12]) between all normally pigmented *S. siderea* colonies and all those with DSS. Further, there were not substantial geographic differences between the Florida and Virgin Island samples. Most previous microbiological work comparing healthy and DSS-affected *S. siderea* has been conducted on corals from the southern Caribbean (i.e., Columbia, Venezuela) [9], [12], providing an opportunity for biogeographic comparisons to our data from Florida and the Virgin Islands.

### Bacterial diversity of healthy *Siderastrea siderea*

This dataset provides the most comprehensive overview to date of the bacterial community associated with the healthy scleractinian coral *S. siderea*. Previous work has consisted of a culture-based study of *S. siderea* mucus [9] and a recent study that employed pyrosequencing [12]. Gil-Agudelo et al. [9] cultured bacteria from *S. siderea* mucus from both healthy and DSS-affected corals. The coral-associated bacterial isolates were identified by metabolic tests (BIOLOG) rather than 16S rRNA sequencing and consisted entirely of Gram-negative Gammaproteobacteria, which probably reflects selection bias of the GASWA culture medium employed [47]. Most metabolic groups were found in both healthy and DSS corals and corresponded to genera identified in this study (e.g., *Vibrio*, *Klebsiella*, *Aeromonas*; Table S1), most notably those in families Enterobacteriaceae and Vibrionaceae (Fig. 3).

Cárdenas et al. [12] used pyrosequencing of the 16S rRNA gene to examine healthy and white plague-affected corals. Their dataset includes 378 OTUs from healthy *S. siderea*. These were dominated by Proteobacteria (75% of sequences), followed by less than 10% each of Firmicutes, Actinobacteria, and Bacteroidetes (in decreasing order of relative abundance) [12]. This perfectly matches the order of relative abundance of our four top phyla (Fig. 2), suggesting the microarray data adequately reflects relative distributions of prominent community members. The remaining phyla detected by pyrosequencing [12] included low percentages of Chloroflexi, Fusobacteria, Verrucomicrobia, Chlorobi, Tenericutes and Cyanobacteria, all of which we also detected (Fig. 2; note that Fusobacteria and Chlorobi were compiled into the ‘other’category). Phyla detected by the PhyloChip G3 in our *S. siderea* samples (Fig. 2) that were not found by Cárdenas et al. [12] include Acidobacteria, Gemmatimonadetes, Planctomycetes and Spirochaetes. This may be due to biogeographical differences between the northern and southern Caribbean, but more likely is due to the greater depth of 16S rRNA sequences queried by the microarray [48].

Two recent clone library studies allowed us to compare *S. siderea* with its sister species from Brazil, *S. stellata* [49], [50]. From these papers we identified 50 sequences, representing 119 clones, from healthy *S. stellata*. At the phylum level, the two species looked similar, with healthy *S. stellata* dominated by Proteobacteria (>80% of sequences), followed by much lower percentages of Bacteroidetes, Cyanobacteria, Actinobacteria, and Verrucomicrobia [49], [50]. However, the two species were markedly different when examined at the family level. Families in common between the two species were Comamonadaceae, Corynebacteriaceae, Flavobacteriaceae, Rhodobacteraceae, and Rhodospirillaceae, as well as Bradyrhizobiaceae, Brucellaceae, Burkholderiaceae, Moraxellaceae, Propionibacteriaceae, Puniceicoccaceae, and Sphingomonadaceae (the latter group being present in *S. siderea* but summed under ‘other’ in Fig. 3) [49], [50]. Families present at very low relative abundance in *S. stellata* but not detected in *S. siderea* were Cytophagaceae and Ruaniaceae [49], [50]. While it is likely that some of the variation seen between these two coral species is due to methodological differences between the studies, we expect that some of the bacterial-community differences are genuine indicators of biogeographic and species differentiation.

### Bacterial diversity of DSS-affected tissues

Gil-Agudelo et al. [9] found a group of bacteria metabolically similar to *Vibrio carchariae* present only in cultures from DSS-affected corals, but subsequent inoculation experiments failed to trigger disease signs. We detected many different *Vibrio* species using the PhyloChip G3, but *V. carchariae* was not one of them (Table S1). There were several OTUs affiliated with *V. campbellii*, *V. harveyi*, and *V. orientalis* that had increased relative intensities in many of the Virgin Island samples compared to those from Dry Tortugas (Fig. S2). Perhaps this is an indication that the Virgin Island corals are more stressed (and therefore conducive to hosting higher abundance of this opportunistic taxon). The Virgin Islands National Park hosts an order of magnitude more visitors per year than the Dry Tortugas National Park (e.g., in the 2009 sampling year, VIIS had 415,847 visitors versus 52,011 in DRTO; NPS Visitor Use Statistics https://irma.nps.gov/Stats). Further, while a large proportion of the VIIS visitors will reach the easily accessible Hawknest Bay, only a small percentage of the visitors to DRTO reach Loggerhead Key since most day trips visit Garden Key alone.

The only previous study to employ molecular techniques in examining DSS-affected corals found significant differences between the bacterial communities in healthy versus affected colonies of *Stephanocoenia intersepta*, both by DGGE and clone library analyses [40]. In contrast, our data did not indicate a significant difference between healthy and DSS-affected corals or between corals from different geographic locations (Figs. 2, 3, 4). This could mean that DSS represents a different condition in *St. intersepta* than in *S. siderea*, as has been previously suggested due to different lesion morphologies [20], [23]. It could also be due to the broader taxonomic coverage achieved using the PhyloChip G3 microarray to examine the coral-associated bacterial communities (4,978 OTUs versus 50 clone library sequences).

Specifically, Sweet et al. [40] found an increase in Cyanobacteria and Actinobacteria associated with all DSS-affected samples and that the Cyanobacteria were dominated by a species of *Oscillatoria*. The genus *Oscillatoria* (reference sequence is *Pseudocillatoria coralii*) is represented by two OTUs out of the 4,978 OTUs shared by all of our samples and was detected in half of our DSS samples (DRTOSSD08, VIISSSD06, VIISSSD09, and VIISSSD10) but none of our healthy (i.e., normally pigmented) samples (Fig. S3). In addition, there were two other cyanobacterial OTUs (53258 – *Phormidium* and 53730 – *Leptolyngbya*) that had higher relative intensities in a subset of the DSS samples (VIISSSD06, VIISSSD07, VIISSSD09, VIISSSD10) but were present in both healthy and DSS samples (Fig. S4). All four of these cyanobacterial genera have been associated with black band disease (BBD) [44], [51], [52] and so clearly have some capacity to negatively impact corals in certain conditions. Unlike BBD, there was no indication of increased sulfate-reducing or sulfur-oxidizing bacterial populations in our DSS samples.

Only two OTUs were unique to DSS and present in more than 50% of (>4) DSS samples in our study: 53656 representing *Chroococcidiopsis*, a genus of Cyanobacteria in the class Oscillatoriophycideae and OTU 7560 representing an unclassified *Vibrio*. Sweet et al. [40] also found two *Vibrio* species that were only present in DSS-tissues, but concluded that, like *V. carchariae* [9], they were probably not directly involved as pathogens. Further, they also detected *Oscillatoria* sp. in lower abundance in one of their healthy samples, leading them to conclude that this cyanobacterium may contribute to the dark pigmentation of DSS in *St. intersepta* but is unlikely to be a pathogenic cause [40]. Our *S. siderea* data corroborate these conclusions (Figs. S2, S3, S4).

Another aspect of the *St. intersepta* study was that different size DSS lesions were sampled and a difference was found in the bacterial communities between small (1–2 cm) and larger (5–10 cm, >10 cm) spots, suggesting a community shift as the lesion aged [40]. Unfortunately, all of our DSS areas would be categorized as small, although many were collected from the edge of a much larger older lesion (i.e., central dead area with algal recruitment; Fig. S1). This sort of purpling along the edge of an older lesion (e.g., one caused by black band disease) has been described as Dark Spot Syndrome Type II [14].

### Fungi

Previous histological work on *S. siderea* colonies from the Bahamas, Little Cayman, Florida Keys, and Puerto Rico described unidentified endolithic fungal cells associated with dark spot lesions [53], [54]. Sweet et al.'s [40] recent discovery of a fungal ribotype consistently associated with DSS in *St. intersepta* that was genetically similar to a fungal plant pathogen (*Rhytisma acerinum*) that causes ‘tar spot’ disease adds some molecular evidence that DSS may be a fungal disease. Unfortunately, we were unable to amplify fungal 18S rRNA from our *S. siderea* samples due to the ITS primers being overwhelmed by coral 18S rRNA. Further, due to the collection and processing methods we followed, it was not possible to examine any of the samples by histology to look for physical signs of fungal infection. Given the uncertainty of whether DSS is a disease or a non-specific stress response to any physical, chemical, or microbiological insult [55], [56], as well as being unclear if these signs represent different conditions in different coral species [20], it is imperative that future studies combine histology with microbiology to link potential causal agents to a specific pathology at the cellular level [57]. Future studies in the vein of Closek et al. [58] may shed more light on what the coral host is doing during these occurrences, providing greater insight into coral immune responses.

## Conclusions

*S. siderea* maintains a geographically-conserved bacterial community with considerable diversity (>600 genera). There is notable overlap between the bacterial community composition of *S. siderea* and its sister species *S. stellata* at high levels of taxonomy.

Our data do not support the hypothesis that DSS is a bacterial disease. There was neither a single dominant bacterial group observed in all DSS samples to indicate a primary pathogen, nor was there a community shift toward a specific or predictable secondarily opportunistic consortium. Future work should focus on determining if this discoloration indicates the same type of lesion across different coral species via histological analyses. Metagenomics also could be used to address the presence of a particular fungal ribotype.

## Supporting Information

Figure S1 **Photos showing examples of dark spot lesions sampled in this study.** A: DRTOSSD08, B: DRTOSSD10, C: VIISSSD08, D: VIISSSD06, E: VIISSSD07, F: VIISSSD10. Samples with the prefix DRTO are from Dry Tortugas National Park. Samples with the prefix VIIS are from the Virgin Islands National Park.
(TIF)

Figure S2 **Vibrionaceae OTUs present in at least two samples are listed with bar lengths representing the relative OTU intensity.** Genus and species names are given for each OTU; when the OTU was unclassified at the genus level, the family name was used.
(XLSX)

Figure S3 **OTU intensity values are displayed in place of samples on a non-metric multidimensional scaling plot (based on a Bray-Curtis similarity matrix) of post-scale normalized data for (A)** ***Oscillatoria*** **_52941 and (B)** ***Oscillatoria*** **_53657 (reference GenBank entry is** ***Pseudoscillatoria coralii*** **).** The intensity values ranged from 12 to>25,000 and are shown in exponential notation; e.g., 3E3 = 310^3^ = 3,000.
(PDF)

Figure S4 **Cyanobacterial OTUs present in at least two samples are listed with bar lengths representing the relative OTU intensity.** Genus and species names are given for each OTU; when the OTU was unclassified at the genus level, the family name was used.
(XLSX)

Table S1 **List of bacterial genera detected in** ***Siderastrea siderea*** **samples based on 4,978 operational taxonomic units (OTUs) detected by the PhyloChip G3 microarray.** OTUs that were unclassified at the genus level are not shown.
(XLSX)
