# Supplementary figures and images for: Comparing Bacterial Community Composition of Healthy and Dark Spot-Affected Siderastrea siderea in Florida and the Caribbean
Source: PLoS One. 2014 Oct 7;9(10):e108767. doi: 10.1371/journal.pone.0108767 (PMC4188562; doi:10.1371/journal.pone.0108767)

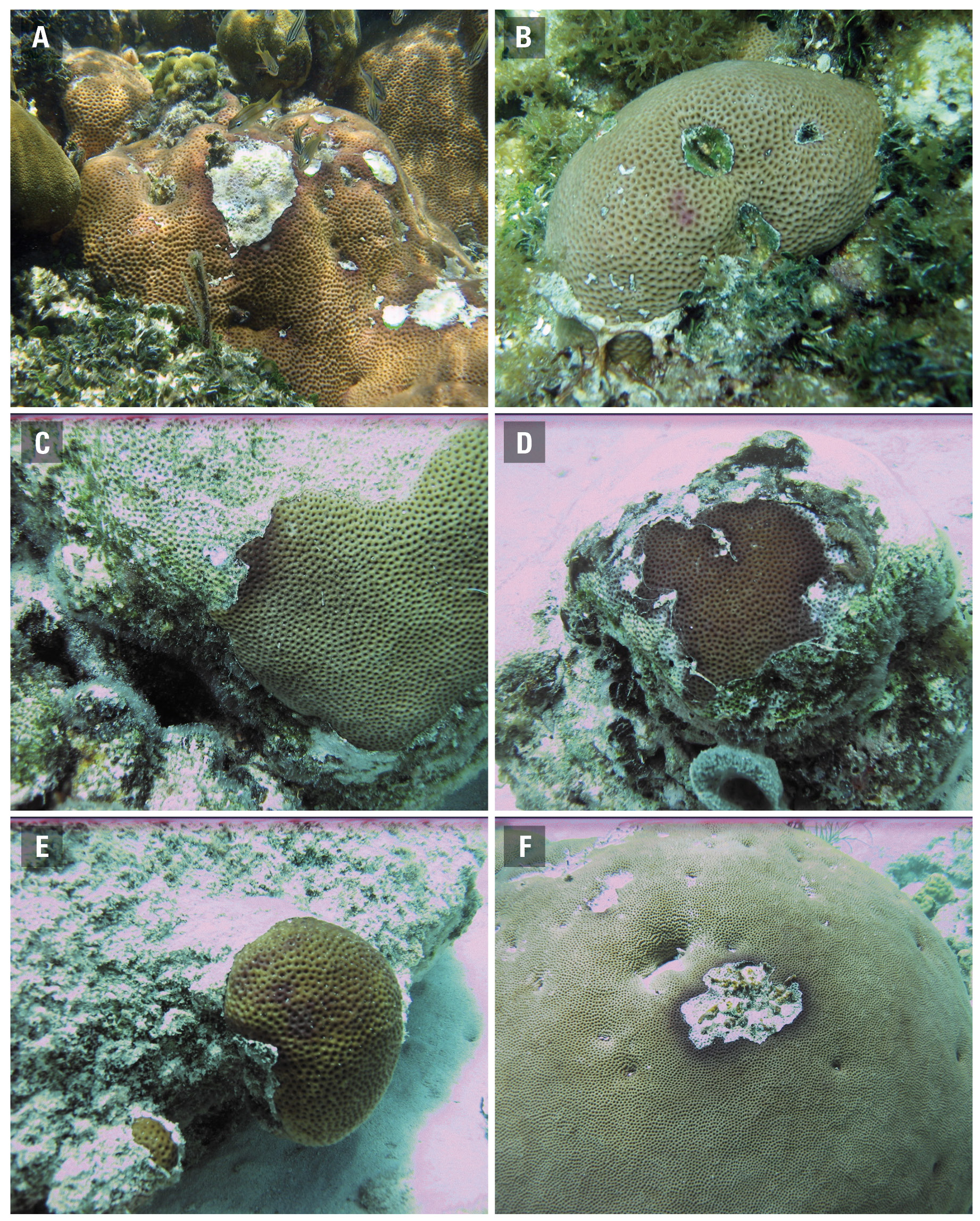

Supplement: Figure S1 — Photos showing examples of dark spot lesions sampled in this study. A: DRTOSSD08, B: DRTOSSD10, C: VIISSSD08, D: VIISSSD06, E: VIISSSD07, F: VIISSSD10. Samples with the prefix DRTO are from Dry Tortugas National Park. Samples with the prefix VIIS are from the Virgin Islands National Park. (TIF) [file pone.0108767.s001.tif]

A)

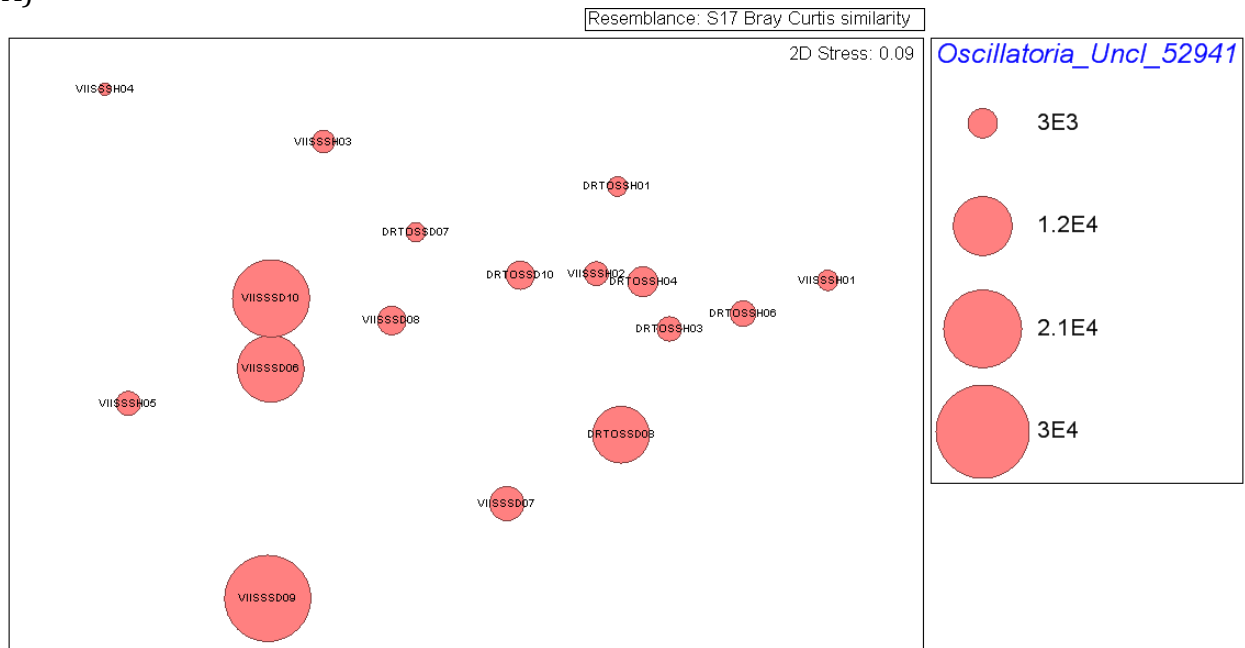

B)

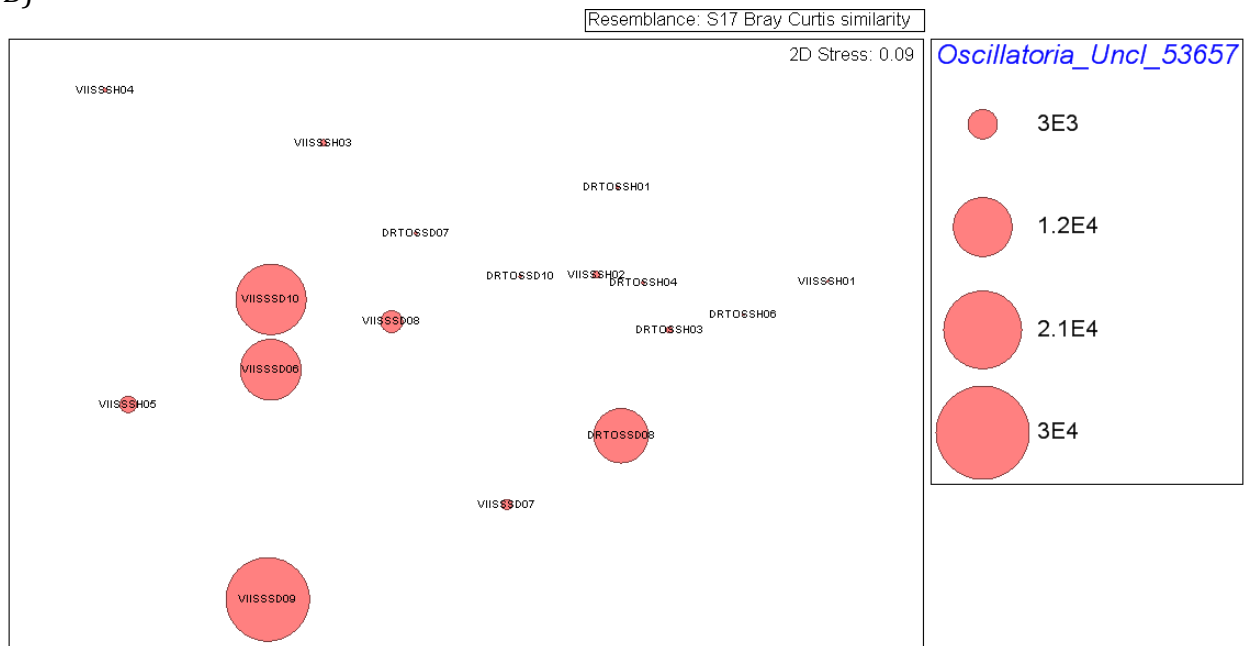

Supplement: Figure S3 — OTU intensity values are displayed in place of samples on a non-metric multidimensional scaling plot (based on a Bray-Curtis similarity matrix) of post-scale normalized data for (A) Oscillatoria _52941 and (B) Oscillatoria _53657 (reference GenBank entry is Pseudoscillatoria coralii ). The intensity values ranged from 12 to>25,000 and are shown in exponential notation; e.g., 3E3 = 3103 = 3,000. (PDF) [file pone.0108767.s003.pdf]
